# First successful outcomes of pegvaliase (PALYNZIQ) in children

**DOI:** 10.1186/s12920-024-01847-1

**Published:** 2024-03-21

**Authors:** Majid Alfadhel, Rayyan Albarakati

**Affiliations:** 1 College of Medicine, King Saud bin Abdulaziz University for Health Sciences (KSAU-HS), King Abdulaziz Medical City, Ministry of National Guard Health Affairs (MNG-HA), Riyadh, Saudi Arabia; 2 Genetics and Precision Medicine Department (GPM), King Abdullah Specialized Children Hospital (KASCH), King Saud Bin Abdulaziz University for Health Sciences, King Abdulaziz Medical City, Ministry of National Guard Health Affairs (MNG-HA), Riyadh, Saudi Arabia; 3 Medical Genomic Research Department, King Abdullah International Medical Research Center (KAIMRC), Ministry of National Guard Health Affairs (MNG-HA), Riyadh, Saudi Arabia

**Keywords:** Phenylketonuria, Pegvaliase, PALYNZIQ®, Pediatric, Case Report, Phenylalanine hydroxylase, PAH, PKU

## Abstract

**Background:**

PKU is an autosomal recessive hereditary inborn error of metabolism caused by a lack of phenylalanine hydroxylase enzyme activity. Pegvaliase (PALYNZIQ®) treatment has been approved to reduce blood Phe concentrations in adult phenylketonuria patients with uncontrolled blood Phe concentrations greater than 600 micromol/L on current management. However, data regarding individuals under the age of 16 is still unavailable.

**Case report:**

We report a 12-year-old Saudi girl who underwent pegvaliase therapy and was closely monitored for one year. Remarkably, a positive therapeutic response became apparent six months after commencing pegvaliase treatment. Phenylalanine (Phe) levels showed significant improvement, stabilising within the < 5 to 14 µmol/L range on a regular diet without any restriction. At her current age of 12, the patient maintains an unrestricted dietary regimen, consuming a diverse selection of foods, including poultry, meat, and protein sources, all while consistently maintaining normal Phe levels with no change in mental status after treatment. The parents gave their written, informed consent in allowing the research study to be carried out and clinical data to be published.

**Conclusions:**

This report addresses the potential broader applications of Pegvaliase in children, as well as its safety and tolerability in this age group. However, larger sample sizes and robust methodologies are required to validate such findings.

## Introduction

Phenylketonuria (PKU) is an autosomal recessive hereditary metabolic disorder caused by a deficiency in the activity of phenylalanine hydroxylase (PAH; EC 1.14.16.1). This deficiency leads to an interruption in the enzymatic conversion of phenylalanine (Phe) into tyrosine (Tyr) within the liver, resulting in elevated Phe concentrations both systemically and in the brain [1–3].

The consequences of elevated blood Phe levels encompass deficits in executive cognitive functions, behavioral abnormalities, and psychiatric disturbances, including depression and anxiety. Furthermore, these effects extend to compromised quality of life (QoL), disrupted mood regulation, and impaired attention span [2, 4–7]. The clinical spectrum of PKU ranges from mild hyperphenylalaninemia, characterised by blood Phe levels predominantly below 360 µmol/L, to the more severe classical PKU phenotype where untreated Phe levels in the bloodstream exceed 1200 µmol/L [1].

The timely management of blood Phe levels through dietary interventions has effectively reduced the occurrence of severe neurological complications during the critical phase of childhood brain development. However, significant medical challenges persist despite these advancements [8]. Sapropterin dihydrochloride (KUVAN®), a synthesised 6R-tetrahydrobiopterin (BH4) co-factor, historically represented the sole pharmacotherapeutic option for BH4-responsive PKU cases. This treatment primarily addresses milder phenotypic presentations characterised by residual PAH activity [9, 10]. A paradigmatic advance emerged in May 2018 with the FDA’s endorsement of pegvaliase (PALYNZIQ®, BioMarin Pharmaceutical Inc., Novato, CA, USA), a novel enzyme substitution therapy for adult PKU patients whose blood Phe concentration exceeds 600 µmol/L. In May 2019, the European Commission approved pegvaliase for PKU patients aged 16 years or older with inadequate blood Phe control [11]. The pegvaliase clinical trials signify significant progress in advancing the PKU therapeutic landscape [12–18]. Substantial reduction in blood Phe levels has exhibited correlations with sustained enhancements in neuropsychiatric outcomes, a phenomenon observed over the course of long-term pegvaliase treatment. The objective is for individuals undergoing pegvaliase treatment to attain consistent, lifelong control over blood Phe levels, thereby fostering cognitive and psychosocial improvement while consuming protein-unrestricted diets.

Evaluating adverse events (AEs) associated with pegvaliase treatment in PKU patients provides crucial insights into the therapy’s safety profile. Injection site reactions and arthralgia are the most common AEs, typically displaying a temporary course. Instances of anaphylaxis reactions, although infrequent, tend to be more prevalent within the initial six months of treatment initiation [17, 19].

A limited number of patients aged 16 to 18 underwent pegvaliase treatment, with AEs resembling those observed in adult patients regarding both type and frequency. Conversely, data concerning individuals under the age of 16 remains unavailable. This article presents the first case report detailing a 12-year-old Saudi girl initiated on pegvaliase and closely observed over one year, yielding promising outcomes. This may underscore the rationale for exploring the potential benefits of pegvaliase in the pediatric age group. The reporting of this article aligns with the Case Report Guidelines (CARE) [20].

## Case report

The index case was a 12-year-old Saudi girl, the offspring of healthy, non-consanguineous parents, and she had two elder healthy sisters. The pregnancy transpired without complications, with unremarkable findings during the antenatal ultrasounds. Her delivery occurred at full term (38 + 2 weeks) via cesarean section due to a history of a prior cesarean procedure. Her birth measurements fell within the normal range: length at 53 cm (90th percentile), weight at 3.66 kg (70th percentile), and head circumference at 34.5 cm (25th-50th percentile).

At eight months of age, the parents sought medical evaluation owing to concerns about her global developmental delay. She exhibited an inability to sit or crawl independently. The family pursued medical opinions from various centres. Yet, a definitive diagnosis remained elusive as the patient was born in Buraidah city (a small city in Saudi Arabia) with no neonatal screening program at the time of delivery. Correspondingly, the diagnosis was revealed at three years old when her parents consulted a pediatric neurologist, who observed that her developmental delay was accompanied by speech delay, hyperactivity, fair skin, blond hair, and hypotonia. An initial brain magnetic resonance imaging (MRI) did not reveal any abnormalities.

A comprehensive analysis of plasma amino acids indicated a notably elevated Phe level of 2116 µmol/l (reference range: 37–78 µmol/l) alongside a reduced Tyr level of 30 µmol/l (reference range: 40–92 µmol/l). Subsequently, she was referred to a different genetics institution and subsequent DNA molecular genetic testing uncovered a homozygous pathogenic missense mutation identified as NM_000277.3(PAH): c.754 C > T (p. Arg252Trp) in the *PAH* gene. Following diagnosis, the patient was promptly initiated on a Phe-restricted dietary regimen and prescribed KUVAN® (sapropterin dihydrochloride); however, this intervention did not achieve noticeable clinical improvement.

Referred to our care at the age of 9 years, the patient presented with mild intellectual disability and hyperactivity during her clinical assessment at that time. Regarding developmental milestones, she exhibited the capability to articulate coherent sentences, engage in writing activities, self-feed, and independently manage dressing tasks. Her overall developmental level was functioning at a level akin to a 5-year-old. This observation was corroborated by her assessed intelligence quotient (IQ) of 73, classifying her within the range of mild intellectual disability.

Upon physical examination, her height measured 142.3 cm (> 95th percentile). Her weight registered at 42 kg (90th -95th percentile) while her head circumference measured 52 cm (5th -10th percentile). Notably, the patient exhibited no discernible dysmorphic features, and her systemic examination yielded unremarkable findings. Although the patient exhibited good catch-up growth, it was evident that her learning capabilities and cognitive functions bore certain limitations. As a result, her educational pursuits were channeled into a specialized school environment. Throughout the course of her evaluation at our clinic, the patient remained under a regimen involving sapropterin dihydrochloride at a dosage of 20 mg/kg per day, complemented by a Phe-restricted dietary regimen.

**Fig. 1:**
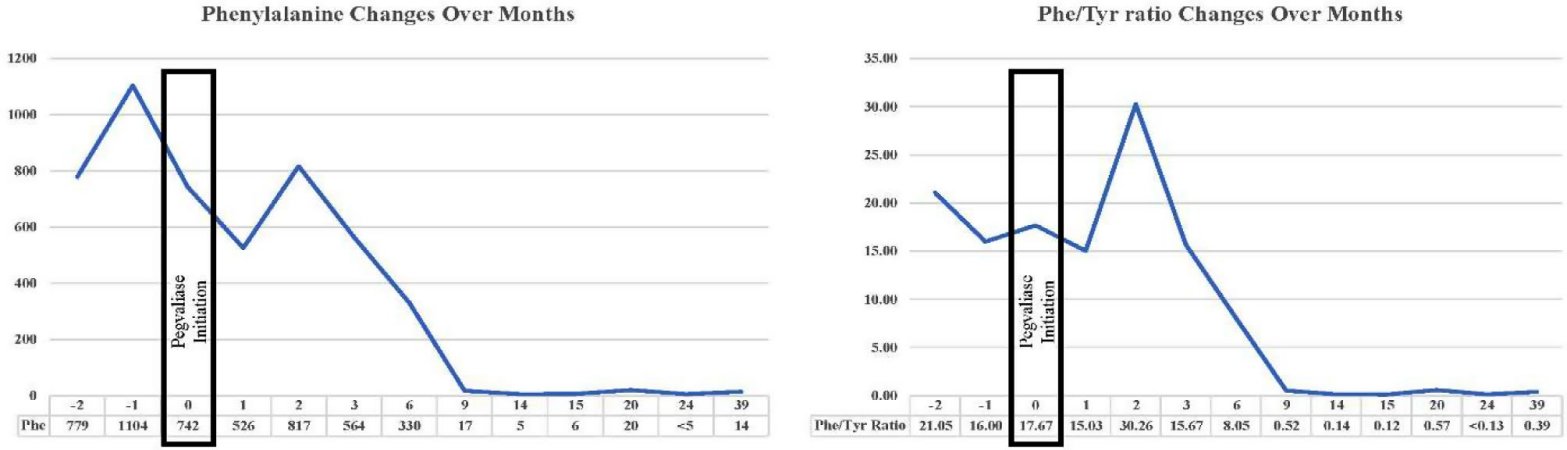
Illustrates the changes over months in Phenylalanine levels (µmol/L) and the Phenylalanine/Tyrosine ratio before and after the initiation of Pegvaliase (titrating dose started from 2.5 mg twice weekly until 20 mg daily)

Over the course of a one-year evaluation, the presented patient struggled with suboptimal control over Phe levels, which exhibited fluctuations ranging between 780 and 1116 µmol/L, (Fig. 1). Consequently, the parents approached us to inquire about the treatment and insisted to start even after we informed them that it was only approved for patients over the age of 16. The parents also asked to begin the treatment on a research basis. As a result, we included the patient in the study. The King Abdullah International Medical Research Centre (KAIMRC) in Riyadh, Saudi Arabia’s Institutional Review Boards (IRB) gave their approval for this study (Ref. RC19/120/R). The parents gave their written, informed consent in allowing the research study to be carried out and clinical data to be published.

Given the challenges in managing her Phe levels, the decision was made to propose the initiation of pegvaliase (PALYNZIQ®) in March 2020, when the patient was 9 years and 3 months old. The therapeutic approach involved a gradual escalation protocol, commencing with 2.5 mg once weekly for the initial month. Subsequently, the dosage was increased to 2.5 mg twice weekly, followed by further increments to 10 mg weekly, 10 mg twice weekly, 10 mg four times weekly, 10 mg daily, and ultimately reaching 20 mg daily.

Throughout this treatment course, consistent monitoring of Phe levels and the Phe to Tyr ratio was undertaken. Preceding the initiation of treatment, the patient’s guardians underwent comprehensive education regarding potential reported AEs and instructions regarding the proper administration of the medication. Emphasis was also placed on the periodic rotation of injection sites to ensure optimal outcomes.

**Fig. 2:**
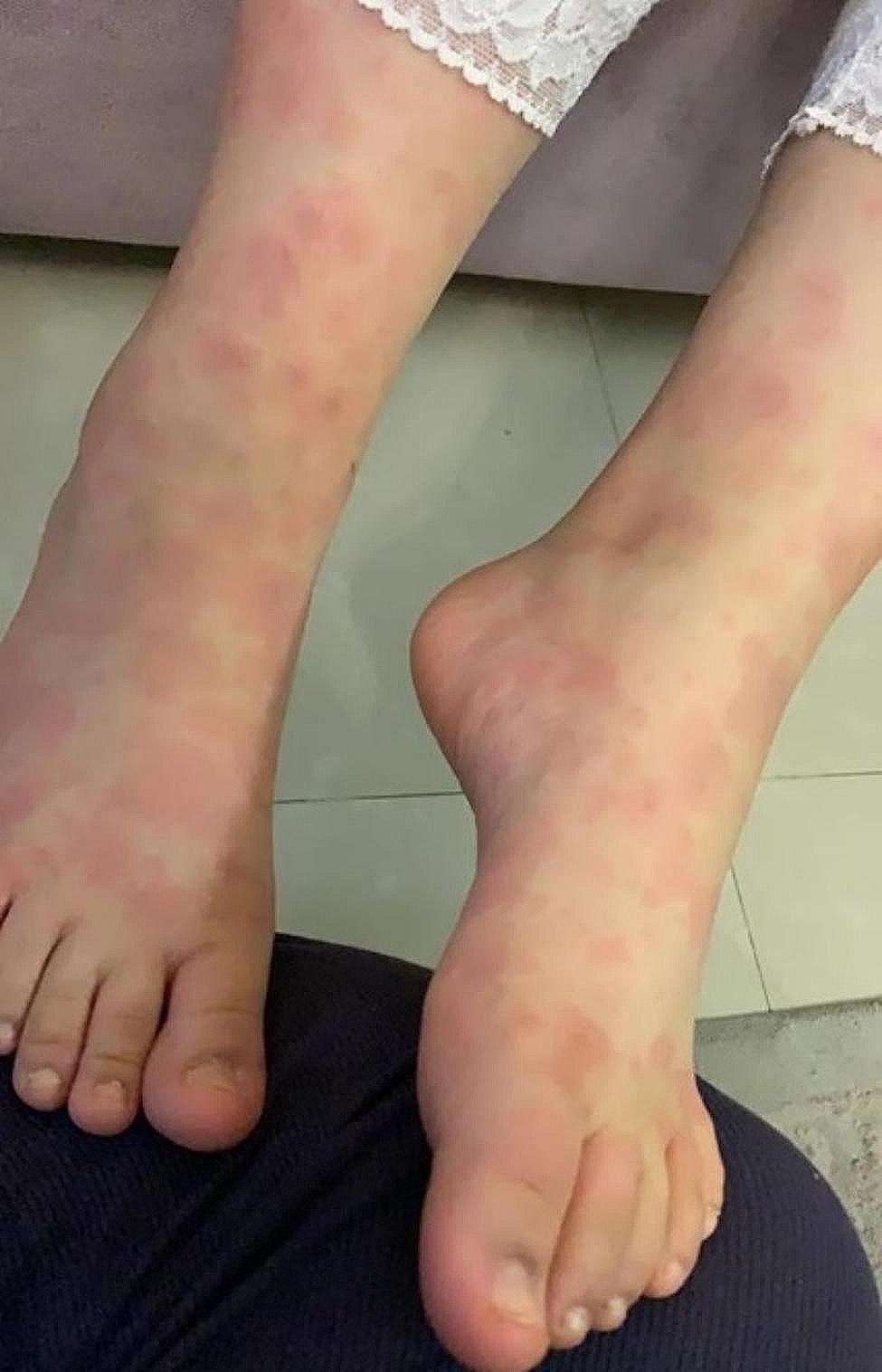
Shows the maculopapular skin rash on the third day after the initiation of pegvaliase

Shortly after commencing treatment, the patient developed a generalised maculopapular skin rash on the third day (Fig. 2). In response, Betnovate Cream was initiated once daily while the treatment course was maintained. The patient did not experience itching, facial swelling, fever, or respiratory distress. The skin rash gradually resolved within 10 days following the initiation of pegvaliase and did not recur thereafter. Approximately six months after initiating pegvaliase treatment, the patient’s responsiveness became evident. The Phe levels showed notable improvement, stabilising within a range of < 5 to 14 µmol/L. Currently, at the age of 12, the patient enjoys an unrestricted diet, consuming various foods, including poultry, meat, and protein, all the while maintaining consistently normal Phe levels with no change in mental status or signs of hypophenylalaninemia.

## Discussion

This article presents this compelling case of first successful usage of Pegvaliasein children, supported by promising outcomes. Despite the absence of prior utilisation of this therapy within this age group, the patient exhibited a remarkable reduction in Phe levels, which fell below 80 µmol/L. This decrease persisted even in instances of non-compliance with the Phe-restricted diet. The Phe levels went down and up after the initiation of our medication, however, after reaching the target of 20 mg/day, the patient consistently maintained Phe levels below 360 µmol/L, along with a favorable Phe/Tyr ratio of approximately one. These positive outcomes remained stable over the subsequent year, and no adverse events (AEs) were reported during this period.

The management approach for PKU revolves around a low-protein diet supplemented by Phe-restricted foods and/or adapted low-protein foods [21, 22]. Nonetheless, this regimen’s complexity challenges adherence and quality of life (QoL) [23]. Individuals with PKU desire innovative therapies to amplify natural protein consumption, diminish reliance on medical foods, bolster mental well-being, and effectively regulate blood Phe levels [24]. Although pegvaliase represents a pioneering treatment that has undergone rigorous clinical trials, none of these trials have encompassed the pediatric age group.

In the initial phase 1 open-label trial (PAL-001, NCT00634660), pegvaliase was evaluated among individuals aged over 18 with classic PKU, with an average baseline Phe concentration of 1310 µmol/L. Over a 42-day follow-up, those receiving the 0.1 mg/kg dose exhibited a noteworthy reduction in Phe levels, with a mean decrease of 48.3% by day six [18].

Pegvaliase dosing strategies were explored through three phase 2 studies. In PAL-002 (NCT00925054), involving 40 adult PKU patients, varied doses (0.001 to 0.1 mg/kg) inadequately controlled blood Phe levels. Similar outcomes emerged in PAL-004 (NCT01212744), a study with 16 adults receiving doses (0.06 to 0.8 mg/kg) five times weekly over 13 weeks. While the regimens in PAL-002 were generally well tolerated, higher doses in PAL-004 correlated with increased hypersensitivity reactions, necessitating dose reduction [25]. In response to these findings, phase 2 trial 165–205 (NCT01560286) was initiated, using an adaptable dosing approach. Twenty-four participants underwent a 4- to 8-week induction phase at 2.5 mg per week, followed by adaptable dose adjustments to maintain blood Phe concentration ≤ 600 µmol/L. After 24 weeks, mean blood Phe < 600 µmol/L by week 11 (56% ± 36% reduction from baseline) was detected. While by 24 weeks of treatment, all achieved < 120 µmol/L [14].

Pegvaliase underwent assessment through a phase 3 study, PRISM-1 (NCT01819727) (17), with 261 participants with 20 mg/d a 40 mg/d group. Baseline Phe averaged 1232.7 µmol/L. Maintenance doses were achieved at around 11.5 weeks (20 mg/d) and 14 weeks (40 mg/d). Notably, Phe levels decreased significantly, by 51.1% at 12 months and 68.7% at 24 months, accompanied by improved inattention and mood scores. AEs were mostly mild to moderate (99%). Few discontinuations occurred due to anaphylaxis and arthralgia. PRISM-1 participants progressed to PRISM-2 (NCT01889862), demonstrating stable Phe levels in the active group, while the placebo group experienced an increase.

This study has limitations, involving a solitary case and a short follow-up duration, raising concerns about generalizability and long-term sustainability. While short-term effects are promising, uncertainties about long-term impact persist, warranting extended follow-up to evaluate its durability on Phe levels and overall well-being. Given PKU’s complexity, future research should explore combining pegvaliase with other therapies and individualised strategies based on precision medicine principles. Investigating these approaches in pediatric PKU patients would address these limitations and refine treatment strategies.

## Conclusion

When considering PKU treatment, patient characteristics and preferences are pivotal considerations. Pegvaliase stands out by lowering blood Phe independently of PAH activity, setting it apart from existing therapeutic options. FDA approval for pegvaliase is exclusive to adults with uncontrolled blood Phe over 600 µmol/L. Across phases 1 to 3, pegvaliase significantly reduces Phe levels, improving inattention and mood; however, these benefits should be weighed against potential AEs. We presented a case of a 12-year-old girl on pegvaliase under close monitoring, showing positive outcomes and a good safety profile. This highlights broader pegvaliase applications, underscoring the need for ongoing research into its safety and efficacy in younger patients.

## Data Availability

The datasets will be available from the corresponding author upon reasonable request.
